# Characteristics of coronary arterial lesions in patients with coronary heart disease and hypertension

**DOI:** 10.1186/s40064-016-2828-7

**Published:** 2016-07-29

**Authors:** Jing-Xia Zhang, Hong-Zhi Dong, Bing-Wei Chen, Hong-Liang Cong, Jing Xu

**Affiliations:** Department of Cardiology, Tianjin Chest Hospital, Jizhao Road, Jinnan District, Tianjin, 300222 China

**Keywords:** Hypertension, Coronary heart disease, Risk factor, Interventional treatment

## Abstract

**Objective:**

The aim of this study was to investigate the correlations between risk factors such as hypertension and the complex degrees of coronary arterial lesions (CAL).

**Methods:**

We selected 462 patients with coronary heart disease (CHD) with confirmed the stenosis (≥50 %) in at least one major coronary artery on coronary angiography and divided them into the “CHD with hypertension” group (CHD-HT, n = 306) and the CHD group (n = 156). The characteristics of CAL and the occurrence of 2-year postoperative major adverse cardiac cerebrovascular events (MACCE) in the two groups were observed.

**Results:**

The mean SYNTAX scores (SS) was higher in the CHD-HT group than in the CHD group (*P* < 0.05). The proportions of complex, calcified, and diffused long lesions in the PCI patients’ target vascular lesions, as well as the total MACCE incidence, were significantly higher in the CHD-HT group than in the CHD group (*P* < 0.05). Logistic multifactor regression analysis showed that age, male sex, hypertension, diabetes, hyperlipidemia, and previous history of myocardial infarction were positively correlated with the SS (*P* < 0.05).

**Conclusions:**

The patients with CHD-HT exhibited complicated and diffused CAL, and arterial hypertension can be considered as a risk factor for the complexity of coronary lesions in patients with ischemic heart disease.

## Background

Epidemiological studies have confirmed the close etiological relationship between the risks of hypertension and coronary heart disease (CHD) (Lawes et al. 2002; McInnes 1995). Hypertension has been proven to be a risk factor of CHD, with significant and independent effects. Hypertension and CHD could jointly promote the occurrence and development of coronary atherosclerosis through mechanisms such as influencing the shearing force of blood flow, the coronary endothelial functions, the permeability of the vascular wall, the adhesive characteristic of platelets, and vascular wall remodeling (Figueiredo et al. 2012; Sipahi et al. 2006; Sudano et al. 2011). In addition, hypertension was demonstrated to be an independent risk factor that could impact cardiovascular death events and prognosis (Frohlich and Susic 2007; Turnbull 2003).

Because of the lack of better objective indicators for evaluating the coronary complexities, previous studies about the correlations between risk factors such as the hypertension and the severity and complexity of coronary arterial lesions (CAL) were few. The SYNTAX score (SS) is a lesion-based angiographic scoring system that was developed to quantify the number, complexity, and location of lesions in patients undergoing percutaneous coronary intervention (PCI). Higher SS are related to more-complex diseases. Therefore, patients undergoing PCI with high SSs are likely to have worse prognosis. The SS is based on anatomical features of CAL and can be used to quantitatively evaluate the complexity of CAL according to anatomical features such as lesion location, severity, bifurcation, and calcification. Although its clinical applications have some limitations such as its inability to determine the characteristics of plaques and coronary flow reserve, a large number of studies have confirmed that the SS has strong clinical practicality when used as a risk assessment tool for coronary revascularization (Farooq et al. 2014; Head et al. 2014; Mohr et al. 2013).

The aim of our study was to analyze the relationships between risk factors (e.g., hypertension, aging, diabetes mellitus, and hypercholestirol) and the complexity of CAL in order to investigate the roles of hypertension and other risk factors in the progression of CHD.

## Methods

### Study population

We selected 462 CHD patients who were admitted at the Tianjin Chest Hospital between January 2010 and June 2010 in order to undergo coronary angiography and had confirmed stenosis (≥50 %) in at least one major coronary artery. The subjects were divided into two groups according to the presence or absence of hypertension as follows: the “CHD combined with hypertension” group (CHD-HT; group A; n = 306 patients; 164 men and 142 women; mean age, 69.6 ± 10.6 years) and the CHD group (group B; n = 156 cases; 108 men and 48 women; mean age, 69.5 ± 10.6 years). Patients with secondary hypertension, rheumatic heart disease, cardiomyopathy, pulmonary heart disease, and permanent pacemaker placement were excluded. This study was conducted in accordance with the declaration of Helsinki. This study was conducted with approval from the Ethics Committee of Tianjin Chest Hospital. Written informed consent was obtained from all participants.

### Sample collection

Samples of peripheral venous blood were drawn from the antecubital vein at admission. Plasma triglyceride, low-density lipoprotein (LDL), high-density lipoprotein, glucose, and creatinine concentrations were measured by using an automated chemistry analyzer (Abbott Aeroset, Minnesota, USA) by using commercial kits (Roche Diagnostics Ltd., Germany).

### SS and angiographic analysis

All of the patients underwent selective coronary angiography by using the Judkins technique, among whom 304 received PCI. The patients were given the standard treatment after hospital discharge. Follow-up through telephone, clinic visit, or angiography was performed from January 2012 to June 2012. CAL that led to ≥50 % diameter stenosis in vessels ≥1.5 mm were scored separately. The cumulative SS, which was prospectively calculated by using the SS algorithm on the baseline diagnostic angiogram, was then calculated by adding the scores (Iqbal et al. 2014; Serruys et al. 2009). Two experienced interventional cardiologists blindly analyzed the SS, and the opinion of a third analyst was obtained. In cases of disagreement, the final judgment was made by consensus. The final score was calculated from the individual lesion scores by the analysts, who were blinded to the procedural data and clinical outcomes.

### Diagnostic criteria

In the “Chinese Hypertension Prevention Guide (2010),” *hypertension* is defined the intake of any antihypertensive drug, a systolic blood pressure ≥140 mm Hg and/or a diastolic blood pressure ≥90 mm Hg. If a patient had a history of hypertension and was currently taking antihypertensive drugs, although the present blood pressures did not reach the aforementioned levels, the patient could also be diagnosed with hypertension. *Hyperlipidemia* is defined in the “China Adult Dyslipidemia Prevention Guide (2007)” according to the characteristics and actual situations of the study populations, under normal diet, with a serum total cholesterol ≥5.72 mmol/L, and associated with or without a serum triglyceride level ≥1.70 mmol/L. *Diabetes* is referred to in the “Chinese Type 2 Diabetes Prevention Guide (2010)” as a random (anytime of the day) blood glucose level of 11.1 mmol/L, a fasting (for at least 8 h) blood glucose level ≥7.0 mmol/L, or 2-h glucose level ≥11.1 mmol/L in a glucose tolerance test. The above-mentioned indicators should be confirmed by repeated monitoring in a different day. *Smoking* was defined if the subject had continuously or accumulatively smoked for 6 months or more.

### Definition of clinical events

Major adverse cardiac cerebrovascular events (MACCE) include nonfatal acute myocardial infarction (AMI), sudden cardiac death, revascularization, and ischemic or hemorrhagic stroke. Coronary angiographic restenosis was divided into in-stent restenosis and in-segment restenosis. In-stent restenosis refers to an inner diameter stenosis >50 % (quantitative coronary angiography), whereas in-segment restenosis refers to stenosis (>50 %) occurring at the vessel segment within the 5-mm border of the stent. *Readmission* was defined as rehospitalized in our hospital or other hospitals because of angina pectoris, heart failure, and revascularization.

### Statistical methods

The SPSS17.0 statistical package was used for the statistical analysis. The counting data were expressed as percentages and analyzed by using the *χ*^2^ test. The measurement data were expressed as mean ± standard deviation ($\overline{x}$ ± s) values. The *t* test was used to compare the mean values between the two groups, and the Kaplan–Meier method (log-rank test) was used to compare the event-free survival rate between the two groups, for which survival curves were drawn. The logistic regression model was used in the multivariate analysis, with a *P* < 0.05 considered as statistically significant. With regard to the evaluated parameters, the values distributed were according to a normal distribution.

## Results

### Baseline characteristics

The proportion of patients with a family history of CHD and/or hypertension was higher in the CHD-HT group than in the CHD group (*P* < 0.05). The proportion of males was higher in the CHD group than in the CHD-HT group (*P* < 0.05). Meanwhile, differences in the prevalences of diabetes, dyslipidemia, history of old myocardial infarction, smoking history, cardiac dysfunction, estimated glomerular filtration rate, and low-density lipoprotein and cholesterol levels were not statistically significant between the two groups (Table 1).

**Table 1:** Basic information of the two groups

Group	Disease type [n (%)]					Cardiac dysfunction level III–IV [n(%)]	LVEF (%)
	SAP	UAP	STEMI	NSTEMI	UACHD		
A (*n* = 306)	20 (6.5)	180 (58.8)	58 (19.0)	18 (5.9)	30 (9.8)	38 (12.4)	59.91 ± 8.63
B (*n* = 156)	10 (6.4)	90 (57.7)	42 (26.9)	8 (5.1)	6 (3.8)	16 (10.3)	60.32 ± 9.39
*t/χ* ^*2*^						1.681	1.854

Group	Previous history [n (%)]			LDL-C (mmol/L)	Fasting blood glucose (mmol/L)	eGFR [ml/(min 1.73 m^2^)]
	MI	Previous PCI	Old cerebral infarction			
A (*n* = 306)	26 (8.5)	12 (3.9)	42 (13.7)	2.73 ± 1.07	6.25 ± 1.86	92.37 ± 19.38
B (*n* = 156)	6 (3.8)	6 (3.8)	12 (7.7)	2.68 ± 1.13	6.27 ± 2.01	92.20 ± 20.29
*t/χ* ^*2*^	3.466	0.002	3.644	0.557	0.088	0.279

Group	Age	Male [n (%)]	Other complications [n(%)]		Family history [(%)]	Smoking [n(%)]
			Diabetics	Hyperlipidemia		
A (n = 306)	69.6 ± 10.6	164 (53.6)	82 (26.8)	124 (40.5)	94 (30.7)	122 (39.9)
B (n = 156)	69.5 ± 10.6	108 (69.2)^a^	34 (21.8)	62 (39.7)	24 (15.4)^a^	68 (43.6)
*t/χ* ^*2*^	0.307	8.265	2.302	0.031	14.439	0.591

Group	blood pressure levels	Medications		
		Statin	ACEI or ARB	anti-platelet agents
A (n = 306)	149.3 ± 21.1	65 (21.2)	112 (36.6)	19 (55.2)
B (n = 156)	129.0 ± 12.4^a^	31 (19.8)	11 (7.1)^a^	77 (49.4)
*t/χ* ^*2*^	11.039	0.118	46.185	1.430

A: CHD-H group; B: CHD group

*SAP* stable angina pectoris, *UAP* unstable angina pectoris, *UACHD* untypical or asymptomatic CHD, *OMI* old myocardial infarction, *STEMI* acute ST-elevated myocardial infarction, *NSTEMI* non-ST-elevation acute myocardial infarction, *LVEF* left ventricular ejection fraction, *eGFR* estimated glomerular filtration rate, *LDL-C* low-density lipoprotein cholesterol, *PCI* percutaneous coronary within intervention

Compared with group A, ^a^ *P* < 0.01

### CAL characteristics

The ratios of single-vessel lesion, two-vessel lesion, three-vessel lesion, and left main stem lesion showed no statistically significant differences between the two groups. The mean SS and the proportions of patients with complex lesions, calcified lesions, and diffused long lesions were higher in the CHD-HT group than in the CHD group (*P* < 0.05). The PCI surgery rate did not statistically significantly differ between the two groups. The mean number patients with coronary stenting was higher in the CHD-HT group than in the CHD group (*P* < 0.05, Table 2).

**Table 2:** Characteristic of CAL of the two groups

Group	Single-vessel lesion [n (%)]	Two-vessel lesion [n (%)]	Three-vessel lesion [n (%)]	Left stem artery involved [n(%)]	SYNTAX integration	PCI therapy [n (%)]
A (*n* = 306)	86 (28.1)	93 (30.4)	127 (41.5)	38 (12.4)	17.53 ± 9.72	210 (68.6)
B (*n* = 156)	46 (29.5)	57 (36.5)	53 (34.0)	24 (15.4)	15.21 ± 10.92^a^	94 (60.3)

Group	Lesion of target vessel (vessels)	Simple lesion (A + B1) [n (%)]	Complex lesion (B2 + C) [n (%)]	Middle-severe circuity [n (%)]	Middle-severe calcification [n (%)]
A (*n* = 306)	240	100 (41.7)	140 (58.3)	21 (8.8)	54 (22.5)
B (*n* = 156)	120	78 (65)	42 (35)^b^	11 (9.2)	12 (10)^b^

Group	Diffused long lesion [n (%)]	Average target vessels	Average target lesions (cases)	Average stent implanted	Left stem stent [n (%)]
A(*n* = 306)	98 (40.8)	1.36 ± 0.57	1.51 ± 0.65	1.90 ± 0.93	13 (6.2)
B(*n* = 156)	34 (28.3)^a^	1.23 ± 0.47	1.36 ± 0.60	1.66 ± 0.91^a^	6 (6.4)

A: CHD-H group; B: CHD group

*PCI* percutaneous coronary intervention

Compared with the A group, ^a^ *P* < 0.05, ^b^ *P* < 0.01

### Two-year follow-up results

**Fig. 1:**
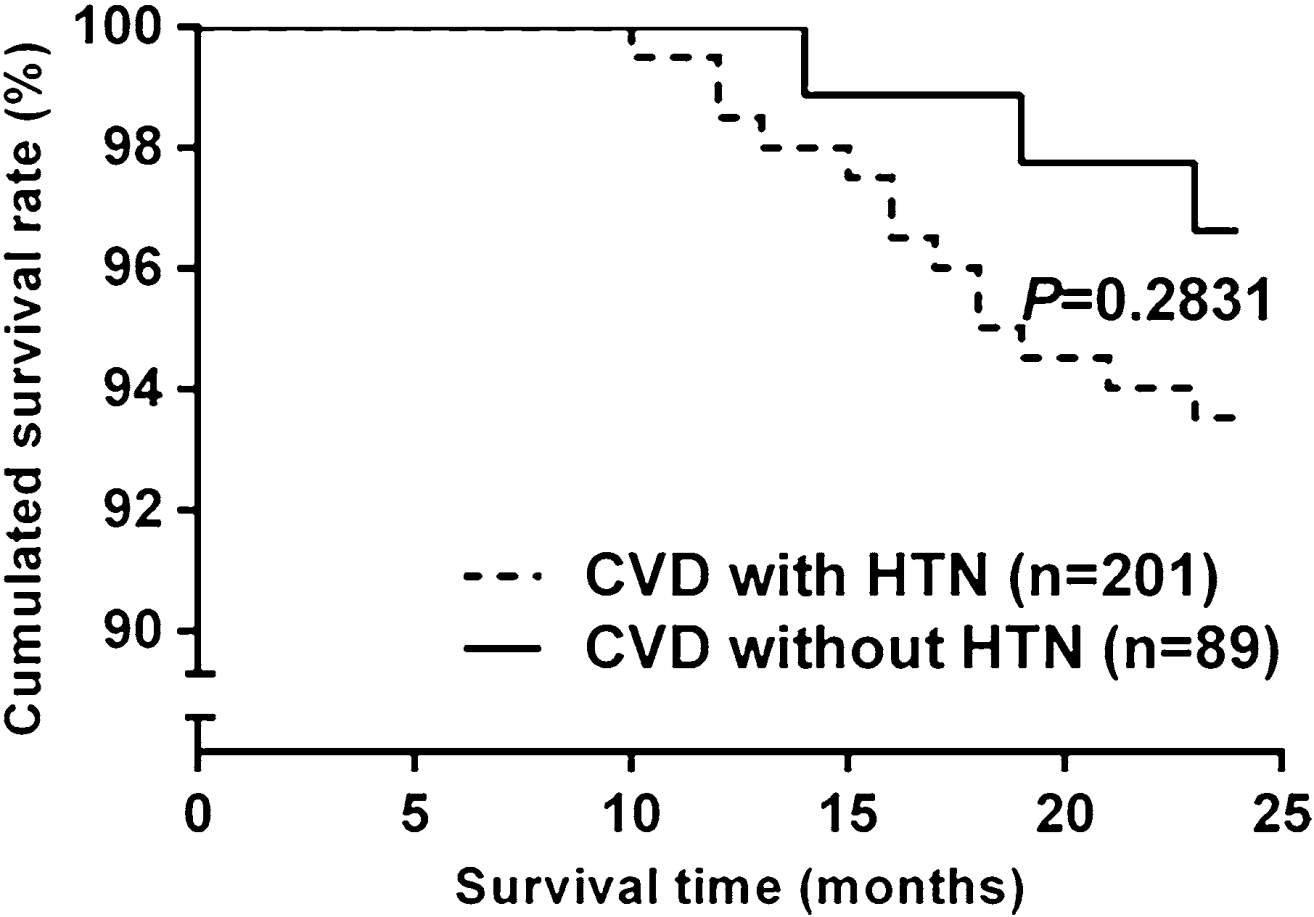
Comparison of event-free survival curves of the two groups

Among the 304 patients who had successful PCI surgeries, 290 (95.4 %) were followed up through telephone calls, 14 (4.6 %) were lost to follow-up, 88 (30.3 %) were followed up through clinic visits, and 34 (11.7 %) were followed up by using angiography. Among the angiographic follow-up cases, 32 patients were followed up by performing coronary angiography within 2 years of PCI because of symptom recurrence and two agreed to undergo follow up with coronary angiography. Although the incidence rates of cardiac death, nonfatal myocardial infarction, re-revascularization, and stroke did not statistically significant differ between the two groups, the overall MACCE incidence was significantly higher in the CHD-HT group than in the CHD group (*P* < 0.05). The CHD-HT group had 16 cases of re-revascularization, among which 5 were caused by non-fatal AMI and 11 were caused by recurrent angina pectoris. The CHD group had 2 cases of re-revascularization, one because of nonfatal AMI and the other because of recurrent angina pectoris. The CHD group had 1 patient with ventricular fibrillation, who survived after the emergency rescue. The difference in all-cause mortality between the two groups was not statistically significant. The long-term rehospitalization and angina recurrence rates were significantly higher in the CHD-HT group than in the CHD group. The >6-month clinical angina remission and in-stent restenosis rates did not statistically significant differ between the two groups (Table 3). The Kaplan–Meier survival analysis showed that the long-term event-free survival rates did not statistically significantly differ between the two groups (*χ*^2^ = 1.152, *P* = 0.2831, Fig. 1).

**Table 3:** Post-PCI 2-year follow-up of the two groups

Group	Cases [n (%)]	MACCE [n (%)]	Cardiogenic death [n (%)]	Nonfatal AMI [n (%)]	Ventricular fibrillation [n (%)]	Re- revascularization [n (%)]	Stroke[n(%)]
A (*n* = 210)	201 (95.7)	35 (17.4)	6 (3.0)	6 (3.0)	0	16 (8.0)	12 (5.9)
B (*n* = 94)	89 (94.7)	6 (6.7)^a^	2 (2.2)	1 (1.1)	1 (1.1)	2 (2.2)	1 (1.1)

Group	All-cause death [n (%)]	Re-hospitalization [n (%)]	Recurrence of angina pectoris[n (%)]	Remission of angina pectoris >6 month [n (%)]	Angiographic follow-up [n (%)]	In-stent restenosis (n)
A (*n* = 210)	13 (6.5)	51 (25.4)	67 (33.3)	141 (70.1)	23 (11.4)	7
B (*n* = 94)	3 (3.4)	9 (10.1)^b^	19 (21.3)^a^	71 (79.8)	11 (12.4)	5

*MACCE* major adverse cardiac cerebrovascular events, *AMI* acute myocardial infarction

Compared with the A group, ^a^ *P* < 0.05, ^b^ *P* < 0.01

### Multivariate logistic regression

In the multivariate logistic regression analysis, age, male sex, hypertension, diabetes, hyperlipidemia, and previous histories of myocardial infarction and smoking were included as covariates into the model. The SS was calculated as a variable (≥22 was defined as 1 and <22 was defined as 0). The results showed that in the general population, the corrected age, sex, hypertension, diabetes, hyperlipidemia, and previous history of myocardial infarction were independent predictors of CHD complexities (*P* < 0.05, Table 4).

**Table 4:** Multifactor logistic regression analysis of general population

	B	SE	Wald	HR (95 % CI)	*P*
Age	1.413	0.301	22.067	4.107 (2.278–7.405)	0.000
Male	0.856	0.292	8.600	2.355 (1.328–4.174)	0.003
Hypertension	0.872	0.309	7.964	2.392 (1.305–4.384)	0.005
Diabetes	0.895	0.289	9.596	2.446 (1.389–4.308)	0.002
Hyperlipidemia	0.915	0.285	10.309	2.497 (1.428–4.365)	0.001
Previous myocardial infarction	1.204	0.449	7.178	3.333 (1.382–8.042)	0.007
Smoke	−0.513	0.298	2.959	0.599 (0.334–1.074)	0.085

Age-adjusted correlation analyses were performed to examine the relationship between systolic blood pressure levels and the SS in the overall population, and in the CHD-HT and CHD groups separately. The results showed that hypertension was significantly related to the SS and systolic blood pressures in the overall population (*P* < 0.05, Tables 5, 6).

**Table 5:** Age-adjusted relations between blood pressure levels and syntax score

Group	B	SE	HR (95 % CI)	*P*
A (*n* = 306)	0.201	0.025	3.630 (0.042 to 0.141)	0.000
B (*n* = 156)	−0.006	0.073	−0.075 (−0.150 to 0.139)	0.325
The whole population	0.165	0.022	3.632 (0.037 to 0.123)	0.000

**Table 6:** correlation analysis of SYNTAX integration and MACCE

	Univariate analysis				Multivariate analysis			
	B	SE	HR (95 % CI)	*P*	B	SE	HR (95 % CI)	*P*
MACCE	6.366	1.542	3.877 (3.140 to 9.593)	0.000	4.062	1.776	2.287 (0.572 to 7.552)	0.023
Cardiac death	9.721	3.609	2.694 (2.629 to 16.813)	0.007	1.825	1.986	0.366 (−7.973 to 11.623)	0.715
Revascularization	10.251	2.405	4.262 (5.524 to 14.977)	0.000	7.169	2.631	2.725 (2.000 to 12.338)	0.007
Nonfatal AMI	3.368	2.823	1.193 (−2.189 to 8.925)	0.234				
Stroke	4.004	2.078	1.927 (−0.086 to 8.093)	0.055				
All-cause mortality	7.759	2.570	3.019 (2.709 to 12.809)	0.003	5.792	3.534	1.639 (−1.152 to 12.736)	0.102

## Discussion

Hypertension is known as a strong risk factor of coronary atherosclerosis. However, whether hypertension is a risk factor of complexity and severity of coronary atherosclerosis remains to be elucidated. In some studies, authors investigated the relationship between the prevalence, severity, and plaque characteristics of coronary atherosclerosis and blood pressure grade. The results of their studies strongly suggest that the incidence of coronary calcification was similarly high in hypertension and diabetes mellitus, and that coronary atherosclerosis shows a grade-response relationship according to hypertension grade (Graham et al. 2012; Grossman et al. 2013; Im et al. 2014; Tomizawa et al. 2015). In this study, the mean SS in the CHD-HT group was higher than that in the CHD group. The analysis of the characteristics of PCI target-vessel lesions revealed that the prevalences of complex, calcified, and diffused long lesions were significantly higher in the CHD-HT group than in the CHD group. The mean number of stent implantations was also significantly higher in the CHD-HT group than in the CHD group, suggesting that the CHD-HT patients had more-complex CAL.

Tanaka et al. (2013) found that among the risk factors of CHD, aging, male sex, and diabetes mellitus were identified as significant independent risk factors of the complexity of CAL. Other coronary risk factors such as hypertension, hypercholesterolemia, and smoking were not identified as significant independent risk factors. While the results of this study were not consistent with the results of their study, this study found that within the general population and the CHD-HT group, not only age, male sex, and diabetes exhibited significantly positive correlations with the SS, but also hypertension, hyperlipidemia, and previous history of myocardial infarction, indicating that hypertension, hyperlipidemia, and previous history of myocardial infarction, as well as age, sex, and diabetes, were independent predictors of the complexity of CAL.

Although the pathogeneses of CHD and hypertension were independent from each other, there existed mutual reinforcing interaction between the two diseases, the atherosclerosis process in CHD patients could be significantly accelerated owing to the presence of hypertension and the reduction in coronary reserve caused by hypertensive microvascular disease could aggravate the coronary arterial stenosis (Delles et al. 2012; Erbel et al. 2012; Juhola et al. 2013; Moges et al. 2014).

Studies have confirmed that hypertension was an independent risk factor of the occurrence and prognosis of CHD events (Makridakis and DiNicolantonio 2014; Nilsson and Cederholm, 2011; Zambon et al. 2014; Zanchetti 2014). In this study, the post-PCI 2-year follow-up results revealed that compared with the CHD group, although the incidence of MACCE (cardiac death, nonfatal myocardial infarction, re-revascularization, and stroke) between the two groups had no statistical significance, the incidence of overall MACCE was still significantly higher in the CHD-HT group than in the CHD group.

Some papers showed an association between coexistence of hypertension and diabetes and negative synergistic effect on SYNTAX score. It may be the diabetes and the hypertension were in the same stage, but there were not the serious symptom. Then the hypertension and diabetes were coexistence. Instead either of hypertension without diabetes or diabetes without hypertension were not associated with high SYNTAX score. These differences may be caused by: First, as mentioned above, different ethnic groups have different lifestyles, eating habits, geographical and cultural differences and other extrinsic factors. Secondly, CHD is multiple factor disease. In addition to the risk factors, the differences existed in gene in different ethnic. The Kaplan–Meier survival analysis revealed that the long-term event-free survival rates between the two groups had no significant difference, indicating that in addition to hypertension, blood glucose and blood lipid levels were also important factors of coronary events and prognosis and that the more-systematic medications of the post-PCI patients might play certain roles in improving the prognosis.

## Conclusions

Arterial hypertension can be considered as a changeable risk factor for the complexity of coronary lesions in patients with ischemic heart disease, and the in-depth understanding of it, as well as the implementation of early therapeutic intervention, could delay CAL progression in CHD-HT patients. Comprehensively controlling various risk factors were extremely important in CHD prevention and prognosis improvements.

## Abbreviations

CAL: coronary arterial lesions
CHD: coronary heart disease
MACCE: major adverse cardiac cerebrovascular events
SS: SYNTAX scores
PCI: percutaneous coronary intervention
LDL: low-density lipoprotein
MACCE: major adverse cardiac cerebrovascular events
AMI: acute myocardial infarction
